# Dio2+Il11+ fibroblasts constitute a local stromal-endocrine niche that promotes intestinal stem cell regeneration after immune-mediated injury

**DOI:** 10.21203/rs.3.rs-11143774/v1

**Published:** 2026-10-16

**Authors:** Pavan Reddy, Takashi Ikeda, Akira Yamamoto, Tyler Leach, Kelli McCord, Lihong Xu, Meng-Chih Wu, Yuichi Sumii, Neelam Bodhale, Yui Kambara, Victoria Ontiveros, Ana Santibanez, Dongchang zhao, Yaping Sun, Laure Maneix, William Hudson

## Abstract

Immune-mediated damage depletes intestinal stem cells (ISCs) and their homeostatic niche. Whether inflammatory injury induces an alternative niche that promotes regeneration remains unknown. Using single-cell spatial transcriptomics in murine gastrointestinal graft-versus-host disease, we identified cytotoxic CD8⁺ T cells accumulated near intestinal crypts coinciding with ISC, Paneth cell depletion, and epithelial death pathway activation. A distinct *Dio2⁺ Il11⁺* fibroblast population emerged in regions of maximal intestinal damage between the epithelial progenitors and stromal cells. These fibroblasts expressed cAMP-response and immune-recruitment programs, suggesting niche reorganization. Thyroid hormone receptor antagonism reduced organoid budding, whereas enhancing thyroid hormone signaling promoted morphogenesis, particularly with IL-11, linking local T3 and IL-11 secretion by these *Dio2⁺ Il11⁺* cells to ISC regeneration. Similar niches were observed in DSS-induced colitis model and in biopsies from patients with Crohn’s disease. Our data identify *Dio2⁺ Il11⁺* fibroblasts as a stromal-endocrine niche at the site of immune injury that supports ISC regeneration.

